# REVIGO Summarizes and Visualizes Long Lists of Gene Ontology Terms

**DOI:** 10.1371/journal.pone.0021800

**Published:** 2011-07-18

**Authors:** Fran Supek, Matko Bošnjak, Nives Škunca, Tomislav Šmuc

**Affiliations:** 1 Division of Electronics, Rudjer Boskovic Institute, Zagreb, Croatia; 2 Bioinformatics and Genomics Programme, Centre for Genomic Regulation (CRG) and UPF, Barcelona, Spain; University of North Carolina at Charlotte, United States of America

## Abstract

Outcomes of high-throughput biological experiments are typically interpreted by statistical testing for enriched gene functional categories defined by the Gene Ontology (GO). The resulting lists of GO terms may be large and highly redundant, and thus difficult to interpret.

REVIGO is a Web server that summarizes long, unintelligible lists of GO terms by finding a representative subset of the terms using a simple clustering algorithm that relies on semantic similarity measures. Furthermore, REVIGO visualizes this non-redundant GO term set in multiple ways to assist in interpretation: multidimensional scaling and graph-based visualizations accurately render the subdivisions and the semantic relationships in the data, while treemaps and tag clouds are also offered as alternative views. REVIGO is freely available at http://revigo.irb.hr/.

## Introduction

Today's high-throughput experiments measure the expression of thousands of genes simultaneously using microarrays, RNA-Seq or various proteomics approaches. ChIP-on-chip or ChIP-Seq experiments are used to determine the genome-wide DNA binding pattern of a specific protein, which may affect a large number of genes. New genomes are being sequenced at an ever-increasing pace and their genes characterized by homology-based annotation transfer. In order to interpret the results of such experiments, statistical testing for over- and under-representation of gene functional categories is used [1]. The formality and structure, along with extensive manual curation, have made Gene Ontology (GO) [2] the vocabulary of choice in these analyses. A multitude of Web servers exists to assist in this task, including but not limited to: L2L [3], FatiGO [4], GOrilla [5] or agriGO [6].

As high-throughput techniques become cheaper and more accurate, they detect even slight changes in gene expression or other measured properties. The lists of relevant genes will grow in size, and so will the derived lists of GO terms. Additionally, the redundancy in the resulting set of GO terms confounds interpretation and inflates the perceived number of biologically relevant results. This is frequently the case when analyzing terms in a parent-child relationship, e.g. the parent term “GO:0009058 biosynthetic process” fully encompasses its child term “GO:0008610 lipid biosynthetic process”. In a list of terms enriched with overexpressed genes, if the child term has highly statistically significant enrichment, the parent term might appear significantly enriched purely as a consequence of including all the genes from the child term.

Thus, a need arises for software that would complement the above-mentioned servers that test for GO category enrichment by starting from their output and providing the facilities for summarizing and visualizing this data. To our knowledge, tools that would assist researchers in interpretation of long GO term lists are scarce, although some Web servers have made a step in this direction, e.g. GOrilla [5] offers a visualization of the enriched GO categories overlaid on the standard GO graph structure. Very recently, a software called RedundancyMiner [7] has been made available that attempts to more directly address the issues of interpretability in GO term lists; we examine its features in more detail in the Results and discussion section below.

In the same vein, researchers may attempt to simplify long GO term lists by replacing the full Gene Ontology with “GO Slims”, cut-down versions of the Gene Ontology. The GO slims are, however, limited to general (high-level) GO terms which are typically less interesting than the more fine-grained terms – the ones that have been removed from the GO slims. Thus, the problem of weeding out the redundant GO terms is not easily solved by removing the GO terms' descendants (or ancestors) in this manner. The complex structure of the GO warrants a solution that takes into account the terms' proximity in the GO graph, quantified by the GO term ‘semantic similarity’ measures [8].

We have implemented a computational approach that (a) summarizes long GO lists by reducing functional redundancies, and (b) visualizes the remaining GO terms in two-dimensional plots, interactive graphs, treemaps or tag clouds. Both the summarization and the visualization step draw on the concept of GO term semantic similarity, reviewed in [8]. In particular, several common measures of semantic similarity [9] that employ the ‘most informative common ancestor’ approach are supported. The implementation is freely available as the REVIGO Web server at http://revigo.irb.hr/.

## Results and Discussion

### A simple algorithm to reduce redundancy within lists of GO terms

Researchers analyzing annotations of gene products are often faced with long lists of GO terms that are either close in the GO hierarchy (sibling terms) or are related by inheritance (child and parent terms). These redundant lists are difficult to interpret, but are likely to contain clusters of semantically similar GO terms.

**Figure 1:**
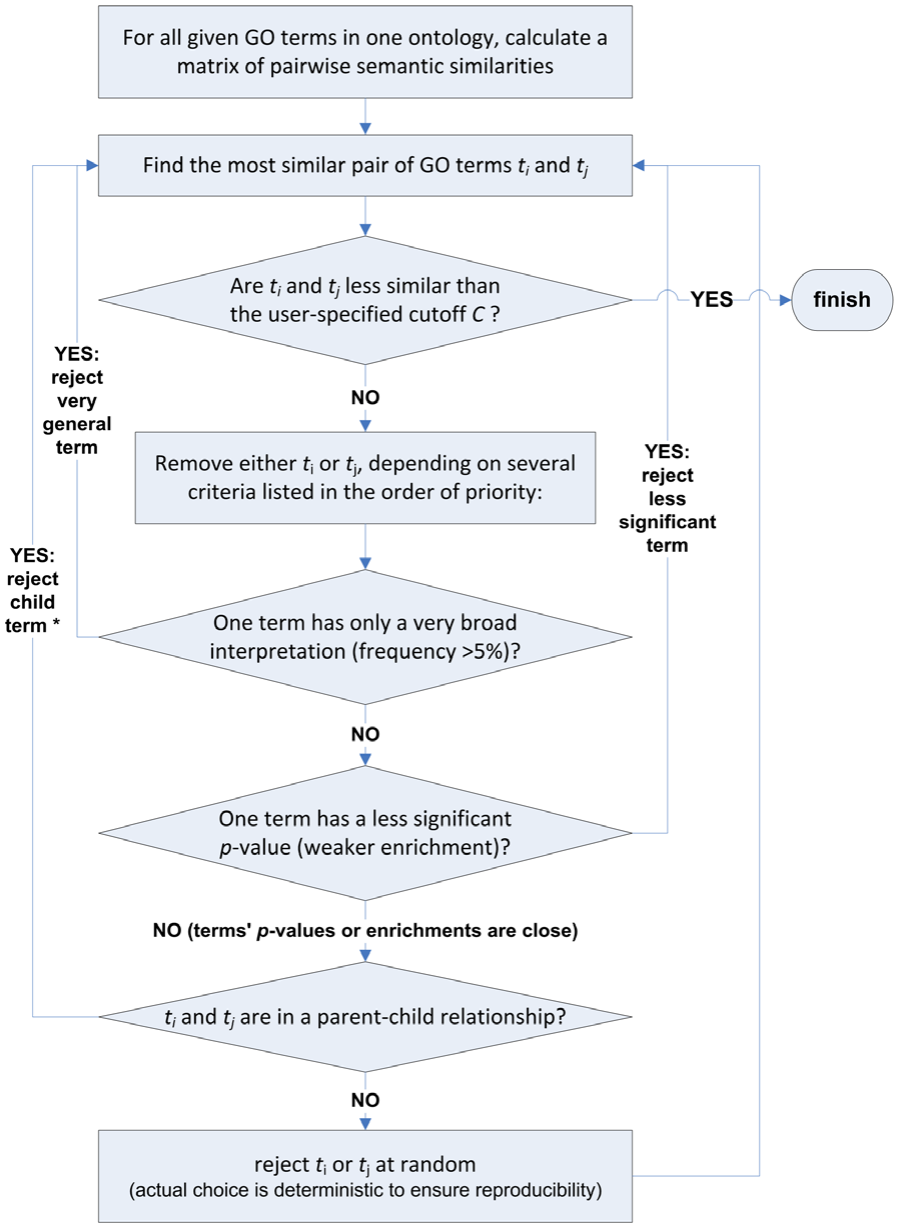
A flowchart describing the REVIGO algorithm to remove redundant GO terms from the provided GO term list. * In a special case when the parent term in question is composed almost exclusively of the child term (>75%), the parent term is rejected instead.

To mitigate the problem of large and redundant lists, we aim to find a single representative GO term for each of these clusters. REVIGO performs a simple clustering procedure which is in concept similar to the hierarchical (agglomerative) clustering methods such as the neighbor joining approach [10]. A flowchart of the steps in the algorithm is given in Fig. 1.

The intuition behind this procedure is to form groups of highly similar GO terms, where the choice of the groups' representatives is guided by the *p*-values, enrichments or similar values that the user supplies alongside the GO terms (Fig. 1). If the *p*-values are quite close and one term is a child node of the other, REVIGO will tend to choose the parent term, with a possible exception when the terms are deemed to be *de facto* equivalent (Fig. 1, see caption). Note that REVIGO generally does not prioritize higher-level or lower-level GO terms as cluster representatives – instead, the user-supplied *p*-values/enrichments are used to guide the selection, if possible. Very general GO terms, however, are always avoided as cluster representatives (Fig. 1) as they tend to be uninformative. It is also possible to manually override the choice of the representative GO term using the ‘pin’ option in case the default solution is not satisfactory for the user e.g. when a more general, higher-level term is desired to represent the group. The user does not necessarily need to provide previously determined *p*-values or another numerical value alongside the GO terms. In that case, REVIGO will prioritize the terms with higher ‘uniqueness’ - the negative of average similarity of a term to all other terms.

The terms that remain in the list after the algorithm has finished are the cluster representatives, where it is guaranteed that no two representatives will be more similar than a user-provided cutoff value *C*. In other words, a lower (more stringent) value of *C* will result in a shorter, but also a more semantically diverse list. To offer some bearing on the relationship of *C* to statistical significance, we conducted a simulation where we drew random pairs of GO terms and recorded the distribution of the SimRel semantic similarity measure [11] (default in REVIGO). One percent of randomly generated GO term pairs have SimRel>0.53. Therefore, at *C* = 0.53 there is a 99% chance an above-background similarity exists between each pair of terms in a cluster. REVIGO offers four pre-defined values of *C* (0.9, 0.7, 0.5 and 0.4) to the user. The lowest value of *C* = 0.4 – corresponding to the “tiny” list size – should be used with caution, as many GO terms might be removed from the list without strong statistical support for their redundancy with respect to other terms. The values of *C* = 0.7 (default) and 0.9 are much more conservative in this respect, but may not shorten the list enough.

### Visualization in scatterplots and interactive graphs

After the clustering procedure described above, the cluster representatives may be submitted to four different visualization procedures: scatterplots, a graph-based visualization, tree maps, and tag clouds.

**Figure 2:**
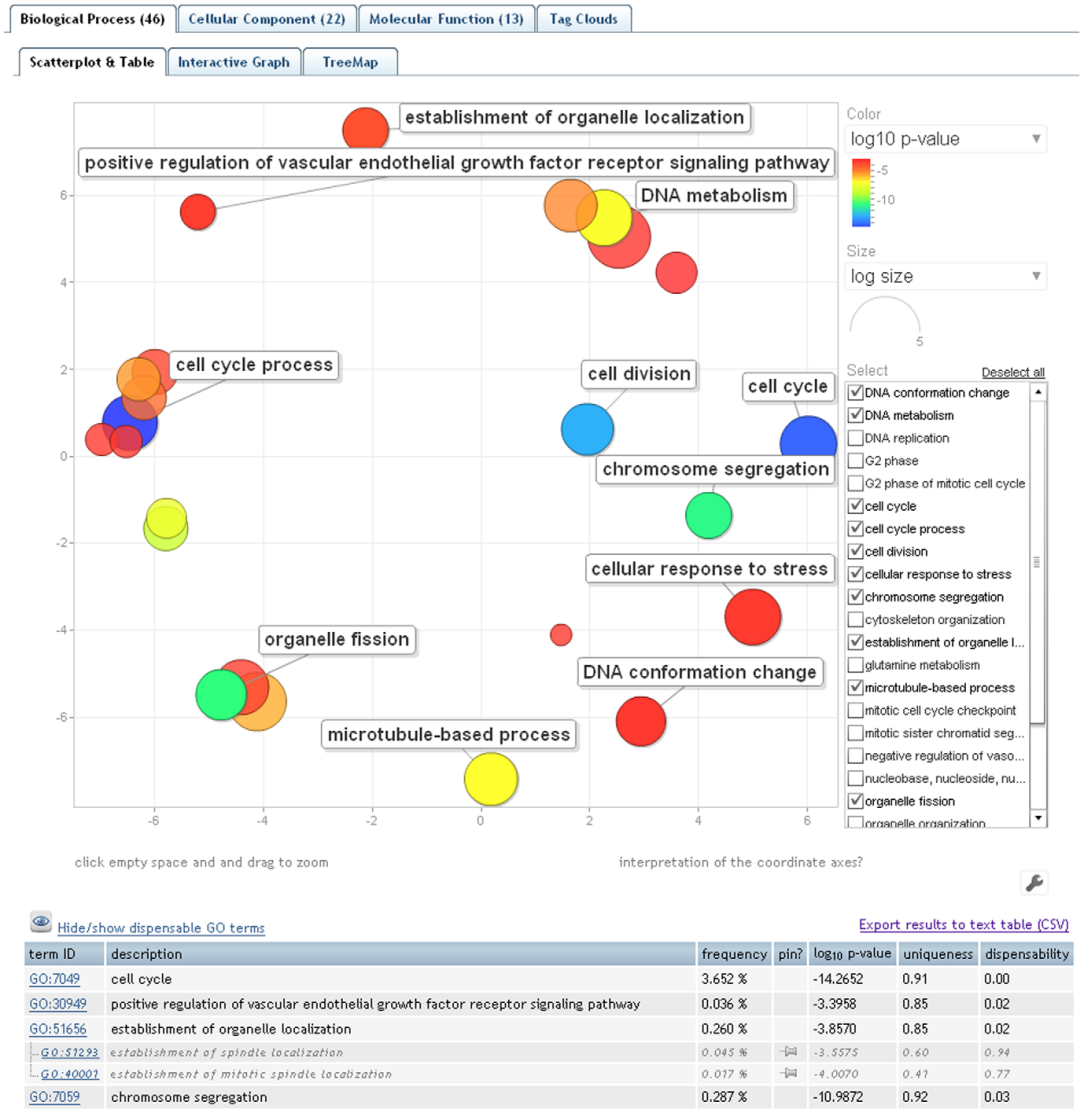
The “Scatterplot & Table” view of REVIGO. The scatterplot shows the cluster representatives (i.e. terms remaining after the redundancy reduction) in a two dimensional space derived by applying multidimensional scaling to a matrix of the GO terms' semantic similarities. The table view in the lower part of the figure is truncated; cluster representatives are given in black and other cluster members in gray letters. Bubble color indicates the user-provided p-value (legend in upper right-hand corner); size indicates the frequency of the GO term in the underlying GOA database (bubbles of more general terms are larger).

In drawing scatterplots (Fig. 2), the challenge lies in assigning *x* and *y* coordinates to each term so that more semantically similar GO terms are also closer in the plot. Here, we employ a multidimensional scaling procedure which initially places the terms using an eigenvalue decomposition of the terms' pairwise distance matrix. This is followed by a stress minimization step which iteratively improves the agreement between the GO terms' semantic similarities and their closeness in the displayed two-dimensional space. The GO terms' and associated data (term descriptions, *p*-values/enrichments, uniqueness, etc.) can be exported to a convenient text table and downloaded.

**Figure 3:**
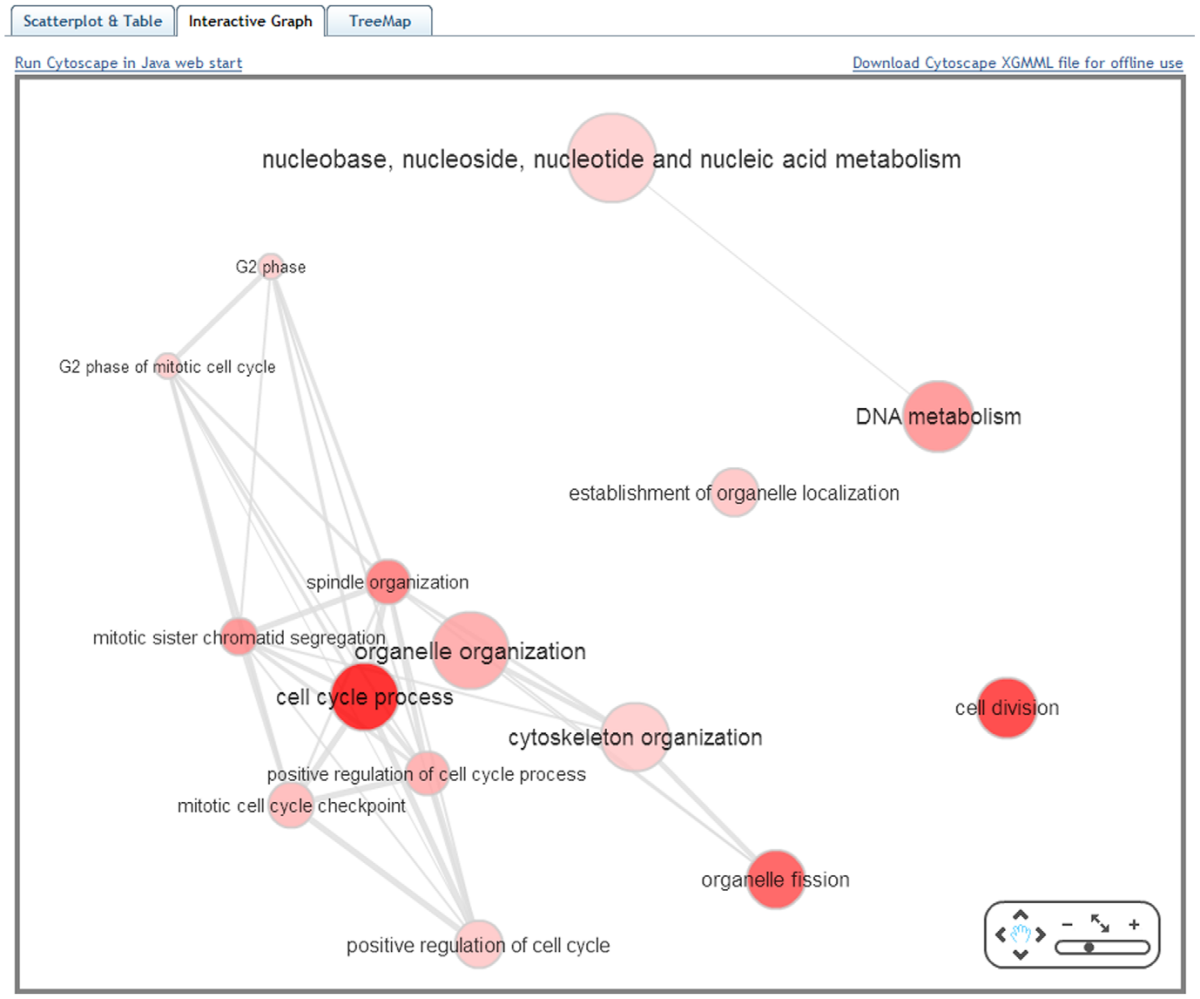
The “Interactive graph” view of REVIGO. Bubble color indicates the user-provided *p*-value; bubble size indicates the frequency of the GO term in the underlying GOA database. Highly similar GO terms are linked by edges in the graph, where the line width indicates the degree of similarity. The initial placement of the nodes is determined by a ‘force-directed’ layout algorithm that aims to keep the more similar nodes closer together, but the placement may later be adjusted by the user.

REVIGO also allows the user to make a graph-based visualization (Fig. 3). Each of the GO terms is a node in the graph, and 3% of the strongest GO term pairwise similarities are designated as edges in the graph. The threshold value of 3% was derived empirically; we found it strikes a good balance between over-connected graphs with no visible subgroups on the one hand, and very fragmented graphs with too many small groups on the other hand. The placement of the nodes is determined by the ForceDirected layout algorithm as implemented in Cytoscape Web [12]. In addition to being viewed in the Web browser, the graph may be exported to a XGMML file, or opened in the stand-alone Cytoscape program [13] via Java Web Start to produce high resolution, publication-quality images. Both visualizations indicate the generality of the GO terms by the bubble radius, where smaller bubbles imply more specific terms; the user-supplied *p*-values/enrichments are shown using color shading.

**Figure 4:**
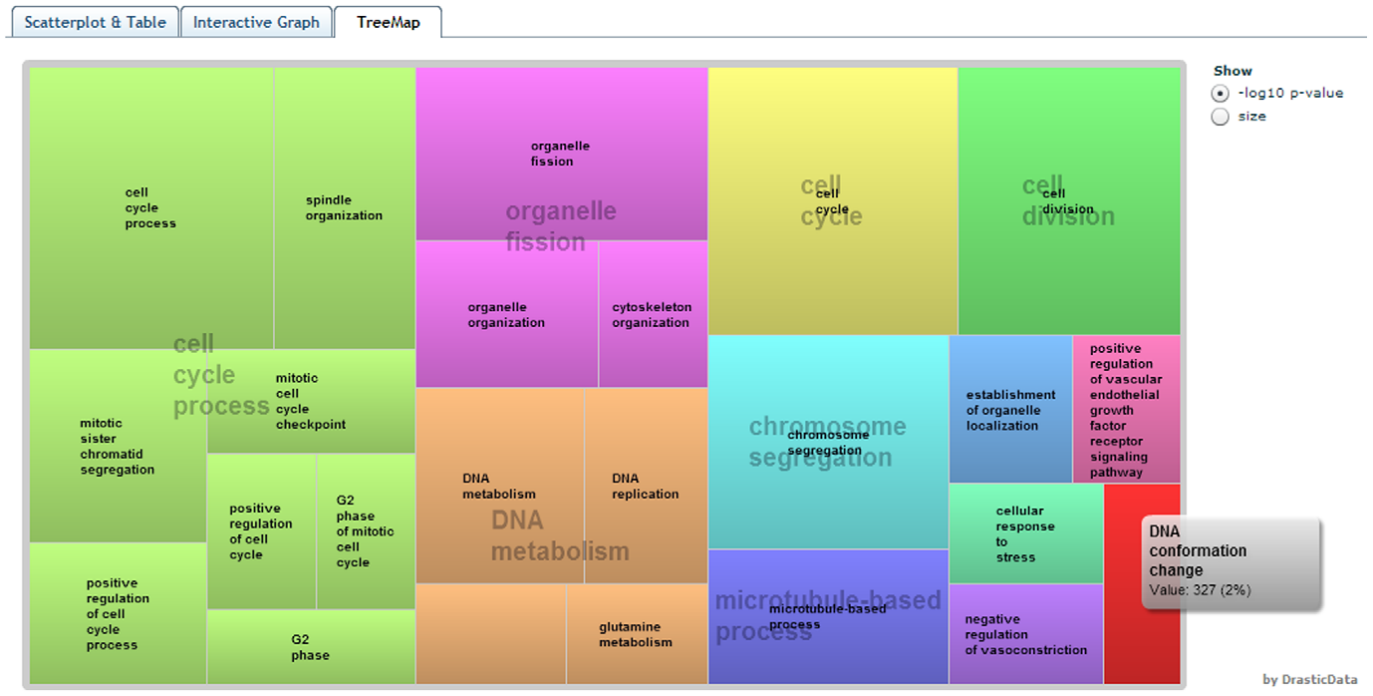
The “TreeMap” view of REVIGO. Each rectangle is a single cluster representative. The representatives are joined into ‘superclusters’ of loosely related terms, visualized with different colors. Size of the rectangles may be adjusted to reflect either the *p*-value, or the frequency of the GO term in the underlying GOA database.

**Figure 5:**
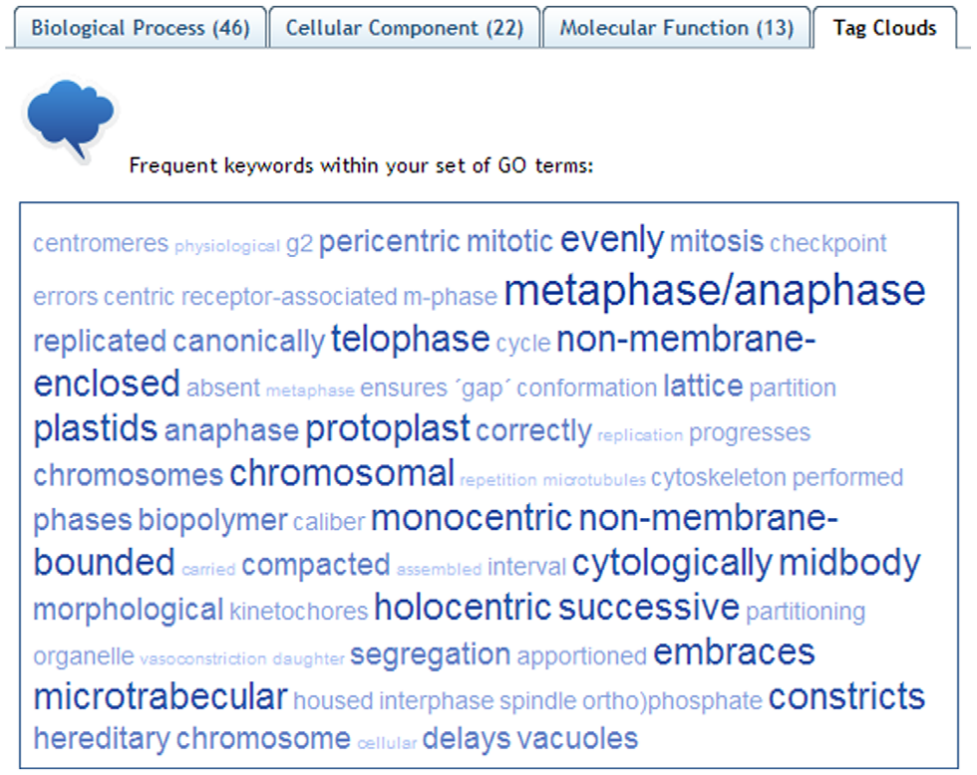
The “Tag Cloud” view of REVIGO. All displayed words are overrepresented in the descriptions of the GO terms in the user-supplied list, with larger and darker letters signifying stronger overrepresentation. Underrepresented keywords are not displayed in the Tag Cloud.

Two additional views of the user's data are supported in REVIGO. Treemaps (Fig. 4) show a two-level hierarchy of GO terms – the cluster representatives from the scatterplot and the graph are here joined into several very high-level groups. Tag clouds show (a) keywords which are overrepresented in the GO terms' descriptions in the GO term list provided by the user (Fig. 5), and also (b) keywords which are correlated to the *p*-values/enrichments supplied by the user.

### An example use-case: summarizing the putative targets of a transcription factor

**Figure 6:**
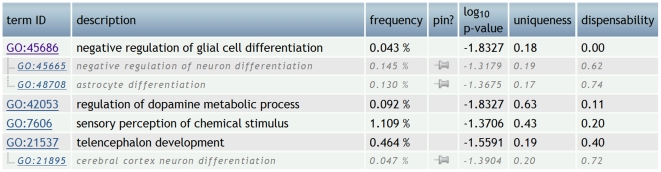
REVIGO's “Table” view of seven gene functional categories co-expressed with the human transcription factor ZNF417; data from [**14**]. “Frequency” is the percentage of human proteins in UniProt which were annotated with a GO term in the GOA database, i.e. a higher frequency denotes a more general term. Cluster representatives are given in black letters and other cluster members are in gray italics and indented. The seven terms are subdivided into four clusters, two of which contain a single term. The ‘pin’ column can be used to manually override the choice of cluster representative.

To illustrate how REVIGO's redundancy elimination algorithm (Fig. 1) works, we turn to a ‘toy example’ which has seven GO categories with associated *p*-values (Fig. 6). This dataset [14] lists gene functional categories co-expressed with the human gene coding for the transcription factor ZNF417, but not with the highly related protein ZNF587, measured using Affymetrix U133plus2 microarrays. The ZNF417 is an evolutionarily recent, great ape-specific transcription factor of which the ZNF587 is a more ancient homolog [14]; gene functions associated specifically to ZNF417 were found to be associated with brain development.

A casual inspection reveals subgroups of redundant gene functions. For instance, the GO term “*cerebral cortex neuron differentiation*” has a high semantic similarity (SimRel = 0.72) to “*telencephalon development*” and is therefore removed by merging it into the cluster represented by the term having a more significant *p*-value (Fig. 6). The removed term is assigned a ‘dispensability’ value of 0.72, a relatively high value reflecting the removed term's strong redundancy with respect to the chosen representative. In the next group of terms, “*astrocyte differentiation*” and “*negative regulation of neuron differentiation*” are similar (0.74 and 0.62, respectively) to “*negative regulation of glial cell differentiation*”. Due to a weaker *p*-value, the first two terms are merged into a cluster represented by the last term (Fig. 6). Note how the choice of cluster representatives is unaffected by whether terms are more general or more specific. The highest remaining pairwise similarity (here, 0.40) is below the user-defined threshold *C*, here set to 0.5, and the clustering algorithm stops. In other words, after having removed the redundant terms, the ones that remain as the cluster representatives are those terms having dispensability values below *C*. The example list of seven GO terms has been reduced to four clusters, of which two are singletons.

A possible alternative for REVIGO's summarization procedure are the frequently used “GO slims”. Here, the seven terms are quite specific and consequently none of them is in the “generic” or “PIR” GO slims (http://www.geneontology.org/GO.slims.shtml). Therefore, the GO slim approach would not apply to this dataset, illustrating the general principle of how summarizing the list by filtering out the more specific (or equivalently, higher information content) GO terms results in a loss of the potentially more interesting results.

In addition to the ‘dispensability’ values, REVIGO provides ‘uniqueness’ values. These two values are anticorrelated, though not perfectly, since ‘uniqueness’ measures whether the term is an outlier when compared semantically to the whole list (without regard for the *p*-values), while the ‘dispensability’ compares a term to other semantically close terms and is assigned based both on the semantic distance and the supplied *p*-values.

**Figure 7:**
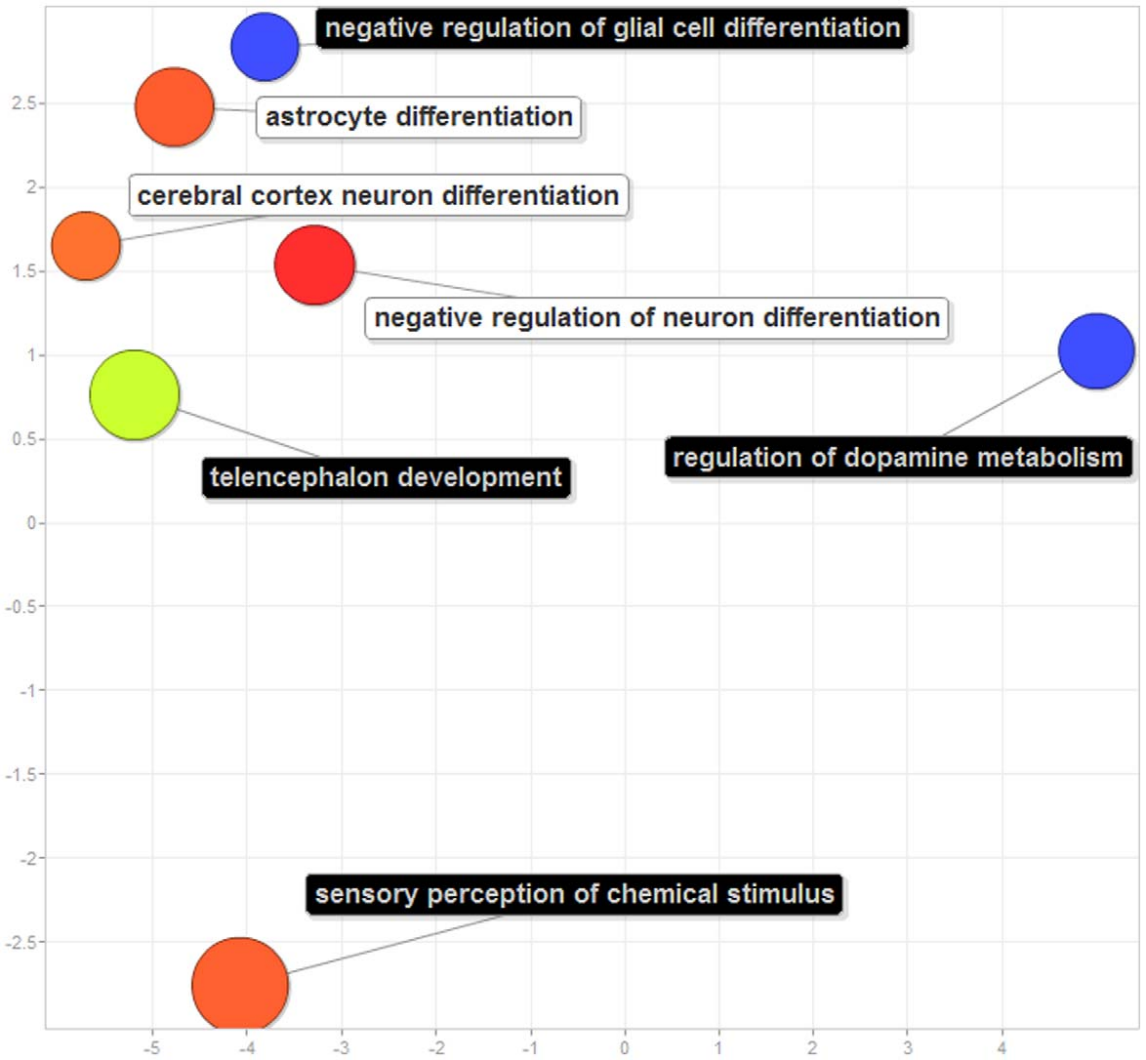
REVIGO's “Scatterplot view” of seven gene functional categories co-expressed with the human transcription factor ZNF417; data from [**14**], also given in **Fig. 6**. Blue and green bubbles are GO terms with more significant *p*-values than the orange and red bubbles. The bubbles' *x* and *y* coordinates were derived by applying multidimensional scaling to a matrix of the GO terms' semantic similarities; consequently, their closeness on the plot should closely reflect their closeness in the GO graph structure i.e. the semantic similarity. The cluster representatives (see legend of Fig. 6) have their description printed on a dark background, while the other cluster members' descriptions have white backgrounds.

To demonstrate the multidimensional scaling-based visualization in REVIGO, we visualize these terms in Fig. 7; for illustrative purposes, all seven terms are visible in this instance, instead of only the four cluster representatives. Here, it can be seen how two terms are quite distinct from the rest and also from each other: “*regulation of dopamine metabolism*” and “*sensory perception of chemical stimulus*” – these terms were not assigned to any of the clusters in the redundancy elimination procedure described above. The remaining five terms are more closely related, where the “*telencephalon development*” and “*negative regulation of glial cell differentiation*” have more significant *p*-values than the three other terms and were thus chosen as cluster representatives.

We provide three ready-to-use examples on the entry Web page of REVIGO. Contrary to the ‘toy example’ described above, these examples contain data a researcher would encounter in a real-life situation: very long, unintelligible lists of GO terms. The data sets cover diverse areas of the life sciences: example #1, a comparative analysis of predicted gene expression levels in bacteria and archaea [15]; example #2, finding putative targets for the cytostatic activity of a small molecule against cancer cell lines [16]; and example #3, gene expression profiling of aggressive breast cancer samples [17]. Data from the example #3 was used to generate the visualizations in Figs. 2, 3, 4, 5.

### A comparison with a related software package

Very recently, software called RedundancyMiner (RM) has been made available [16] that has similar aims as REVIGO – to mitigate the issue of redundancy in lists of GO terms. While it is difficult to quantitatively benchmark and compare programs dealing with subjective categories such as interpretability of a list of GO terms, or the utility of a visualization method in leading to novel insight, we here provide a comparison the features of the two packages. Ultimately, the choice of the best software for a given purpose rests on the user.

- Implementation: RM is a standalone software meant to be installed and run locally, and also requires Perl and a Java virtual machine to be installed. REVIGO is a Web server, meaning no special software needs to be installed on the user's computer prior to use.
- Interoperability: due to the requirement for a specific data input format, RM is tied to the GoMiner package [18] for finding enriched GO terms. REVIGO accepts input from any Web server or software that generates lists of GO terms, and is currently coupled with GOrilla [6] and agriGO [10] to automate the transfer of data.
- Measuring GO term similarity: RM uses a novel approach – a *p*-value of the correlation (by Fisher's exact test) of the genes' assignment to the two categories. This *p*-value-based similarity will appear stronger for two broad (general) GO terms than for two narrow (specific) ones, even if the overlap is the same in both cases. REVIGO by default uses the established SimRel measure of GO term similarity which has been thoroughly benchmarked by examining semantic similarities in GO assignments of homologous proteins [11].
- Threshold for redundancy reduction: In RM, the user can tune the size of the resulting list by selecting the threshold for the Fisher's exact test *p*-value, or the “nominal number of merged pairs” [9] in the list. The range spanned by these quantities is dataset-dependant. In REVIGO, the user chooses the threshold value of SimRel or a related measure [11], which always varies between 0 and 1, and is also independent of the specific dataset, facilitating interpretation and comparison across datasets.
- Overlapping clusters: RM employs a procedure where the same GO term may be assigned to more than one cluster. In contrast, REVIGO always assigns each GO term to a single cluster, thus avoiding a source of redundancy in the results. This may be advantageous since the primary goal of the procedure was to reduce redundancy in the input data.
- Cluster representatives: RM does not explicitly select representatives of the GO term cluster; rather, it labels each cluster using a concatenation of the names of all the GO terms in the cluster. REVIGO uses the user-supplied *p*-values or enrichments to guide the selection of representatives which are well supported by the statistical tests.
- Visualization: as a novel visualization method, RM introduces “Meta-CIMs” (clustered image maps) that show the composition of the GO term clusters. REVIGO offers interactive plots based on semantic similarity and multidimensional scaling, interactive graph visualizations, treemaps and tag clouds.

In addition to RedundancyMiner, in Table 1 we provide an overview of other, more remotely related software. In contrast to REVIGO, these tools perform GO term enrichment analysis i.e. they start from a user-supplied list of genes, but offer additional features to assist in interpretation of the results, typically visualization and clustering facilities.

**Table 1:** Tools that perform GO term enrichment analyses, while additionally offering facilities to assist in the interpretation of results, primarily through visualization.

Tool	Brief description
BINGO [21]	Cytoscape plug-in that tests for GO category enrichment in a list or network of genes, and displays the results in a graph of GO terms.
GOrilla [5]	Web server that tests for GO terms that are significantly enriched at the top of a ranked gene list. Visualizes results overlaid on the standard GO structure.
SimCT [22]	Standalone Java program that performs a hierarchical clustering of a list of gene-GO term annotation pairs. The subtrees of the final clustering are annotated with a relevance score and representative GO terms, and visualized interactively.
Ontologizer [23]	Standalone Java program that supports multiple statistical approaches for testing for GO term enrichment in a list of genes, while compensating for GO term redundancy due to parent-child relationships, including PCU [24] and MGSA [25] methods. Interactive visualization of the results.
GENERATOR [26]	Standalone Windows executable. Uses Non-negative Matrix Factorization to cluster genes into groups with more homogenous GO annotation. Visualizes the clusters at several levels of granularity, together with GO term representatives for each cluster.

REVIGO is the tool of choice for users that wish to be able to quickly analyze their list of GO terms and see if the output fits their needs, without needing to install any software on their machine or to master complex input formats. Furthermore, REVIGO might appeal to users wanting to experiment with different visualization techniques and choose the one best suited to facilitate interpretation of a particular dataset.

### Conclusion

We anticipate REVIGO will be useful to researchers in the life sciences who deal with data from any kind of high-throughput experiments which is subsequently analyzed for overrepresentation in the Gene Ontology functional categories. By relying on semantic similarity measures, REVIGO starts with the output of other software for finding enriched GO terms, forming GO term clusters and displaying only their representatives to ease interpretation by reducing redundancy, while prioritizing the more enriched/statistically significant terms. Several supported modes of visualization allow the researcher to interactively explore the results: for instance, to further group the cluster representatives together into several broad categories, or examine how this grouping relates to the GO terms' generality or their significance in the particular experiment.

## Methods

REVIGO is a server-side Java web application running on a Glassfish 3 application server. For data visualization, REVIGO relies on Google Motion Chart for scatterplots, Cytoscape Web [12] for graphs and DrasticTreemap for treemaps. For multidimensional scaling, the MSDJ library [19] is used.

For calculation of semantic similarity measures between GO terms, REVIGO relies on pre-computed information content (IC) for the GO terms. The IC is calculated as a negative logarithm of the GO term's relative frequency in a reference database – the EBI GOA database [20] – which annotates all UniProt entries with GO terms. The user may optionally decide to select the database with one of the 11 species-specific GOA subsets for common model organisms, in order to fine-tune the calculation of semantic distances (which rely on IC) for the problem at hand. If the particular organism is not offered in REVIGO, the closest available organism or the default UniProt database should generally be adequate replacements, assuming that the relative frequencies of gene functions in the user's genome are not far from the ones in the selected genome, or in case UniProt was selected, from the overall trends in the genomic databases.

REVIGO supports four semantic similarity measures based on the concept of the “most informative common ancestor”: Lin's, Resnik's, Jiang and Conrath's measures, and the SimRel measure [8]. These and other measures and the role of the IC in their calculation are reviewed in [12]. The employed semantic similarity measures are quite robust with regard to future changes in the EBI GOA database due to new or updated annotations, as they don't rely on the GO annotations of each particular gene, but only on the terms' overall IC, which is expected to change little with time. Therefore, an aggressive update schedule is not necessary for REVIGO, and the underlying Gene Ontology and the EBI GOA database will normally be updated on a yearly basis, and more frequently in case of a large-scale release of new GO terms by the GO Consortium.

REVIGO also has a facility for integration with Web servers/software which produce lists of GO categories, typically by testing for statistically significant enrichment of a variable in GO terms; see Introduction for several examples. Owners of such Web servers can use a HTTP POST request to pre-populate REVIGO's input form with output of their server; please refer to the online instructions for technical details.

REVIGO is freely available from http://revigo.irb.hr/. Any modern internet browser with Adobe Flash capabilities is sufficient to access the server; additionaly, client-side Java is required if Cytoscape [13] is invoked via Java Web Start.
